# Cholangiohydatidosis. Clinical features, postoperative complications and hospital mortality. A systematic review

**DOI:** 10.1371/journal.pntd.0011558

**Published:** 2024-03-07

**Authors:** Carlos Manterola, Josue Rivadeneira, Claudio Rojas-Pincheira, Tamara Otzen, Hugo Delgado, Catalina Sotelo, Antonio Sanhueza

**Affiliations:** 1 Center for Morphological and Surgical Studies. Universidad de La Frontera. Chile; 2 PhD. Program in Medical Science, Universidad de La Frontera, Chile; 3 Núcleo Milenio de Sociomedicina. Santiago, Chile; 4 Zero Biomedical Research. Quito, Ecuador; 5 Universidad Católica del Maule, Chile; 6 Pan American Health Organization, Washington, United States of America; Gulu University, UGANDA

## Abstract

**Background:**

Cholangiohydatidosis (CH) is an evolutionary complication of hepatic cystic echinococcosis, associated with increased morbidity and mortality. The aim of this study was to describe the available evidence regarding clinical characteristics of CH, postoperative complications and hospital mortality.

**Methodology/Principal findings:**

Systematic review. Studies related to CH with no language or publication restriction were included. Sensitive searches were performed in Trip Database, SciELO, BIREME-BVS, WoS, PubMed, EMBASE and SCOPUS. MeSH and free terms were used, including articles up to April 2023. The main outcome variables were postoperative complications and hospital mortality; the secondary ones were publication year, origin and design of primary studies, main clinical manifestation, anatomical location and type of cysts, hospital stay, surgical procedure performed, reinterventions; and methodological quality of primary studies, which was assessed using MInCir-T and MInCir-P scales. Descriptive statistics, calculation of weighted averages and their comparison by least squares logistic regression were applied. 446 studies were retrieved from the searches performed, 102 of which met the inclusion and exclusion criteria. The studies analyzed represent 1241 patients. The highest proportion of articles was published in the last decade (39.2%). Reports are mainly from Turkey (28.4%), Greece (9.8%), Morocco and Spain (8.8% each). With a weighted mean of 14.3 days of hospital stance; it was verified that 26.2% of patients developed postoperative complications (74,3% Clavien y Dindo III y IV), 6.7% needed re-interventions, and 3.7% died. When comparing the variables age, postoperative complications, hospital mortality, and reinterventions in two periods of time (1982–2006 vs. 2007–2023), no statistically significant differences were found. When applying the MInCir-T and MInCir-P scales, the methodological quality of the primary studies was 9.6±1.1 and 14.5±4.3 points, respectively.

**Conclusion/Significance:**

CH is associated with severe postoperative complications and significant hospital mortality, independent of the development of therapeutic support associated with the passage of time.

## Author summary

Cystic echinococcosis (CE) is a zoonotic neglected tropical disease which predominantly affects poor pastoral communities globally. The parasite cycles between farm dogs and livestock and is associated with livestock farming and feeding of infected offal to dogs. Human morbidity and mortality occur due to cyst formation in body organs, especially in the liver. One of the evolutionary complications of hepatic CE is the passage of parasitic structures, characteristic of CE (germ or pieces thereof, daughter vesicles or both), inside the bile duct, causing total or intermittent biliary obstruction, with or without secondary infection which is known as cholangiohydatidosis (CH), considered the most frequent evolutionary complication of CE, associated with increased morbidity and mortality, and worsened prognosis of the disease. This review aims to describe the available evidence regarding clinical characteristics of CH, postoperative complications and hospital mortality.

## Introduction

*Echinococcus granulosus* and its various genotypes (preferably G1-G3 and G6-G7), is the etiological agent of cystic echinococcosis of the liver (CEL). This infection manifests itself three weeks after ingesting the egg and can reach 2 to 3 cm. in diameter in the liver parenchyma, three months after the infection occurred [1,2].

As CEL progresses, intracystic pressure reaches values of up to 80 cm H_2_O, producing atrophy and fibrosis of the liver parenchyma due to compression which eventually leads to biliary tree obstruction. This occurrence happens namely in those cysts located in the center of the liver. Similarly, the increase in intracystic pressure and the fragility of the cyst wall, can generate a rupture of the cyst towards the lumen of the bile ducts, developing a cysto-biliary communication (CBC); However, even if there is no cyst rupture into the bile ducts, it can manifest biochemically and radiologically as an obstructive syndrome of the bile duct, secondary to compression [3].

Consequently, the evidence suggests that the most frequent evolutionary complication of CEL, is CBC development, with a reported incidence of up to 42% [4–6]. These communications, in relation to the diameter, can be classified as minor (or simple) and major (or frank intrabiliary rupture). Minor ones, which affect between 10% and 37% of patients with CEL, are small communications between the cyst wall and the bile ducts, where there is no passage of daughter vesicles or pieces of germinative membrane to the bile ducts. Nevertheless, in these cases fluid, protoscolex, or hydatid grit may enter the bile ducts without causing obstruction. Being asymptomatic, pre-surgical diagnosis is unlikely and minor CBCs are detected during surgery, by visualizing bile leakage or bile fistulas [6].

**Fig 1:**
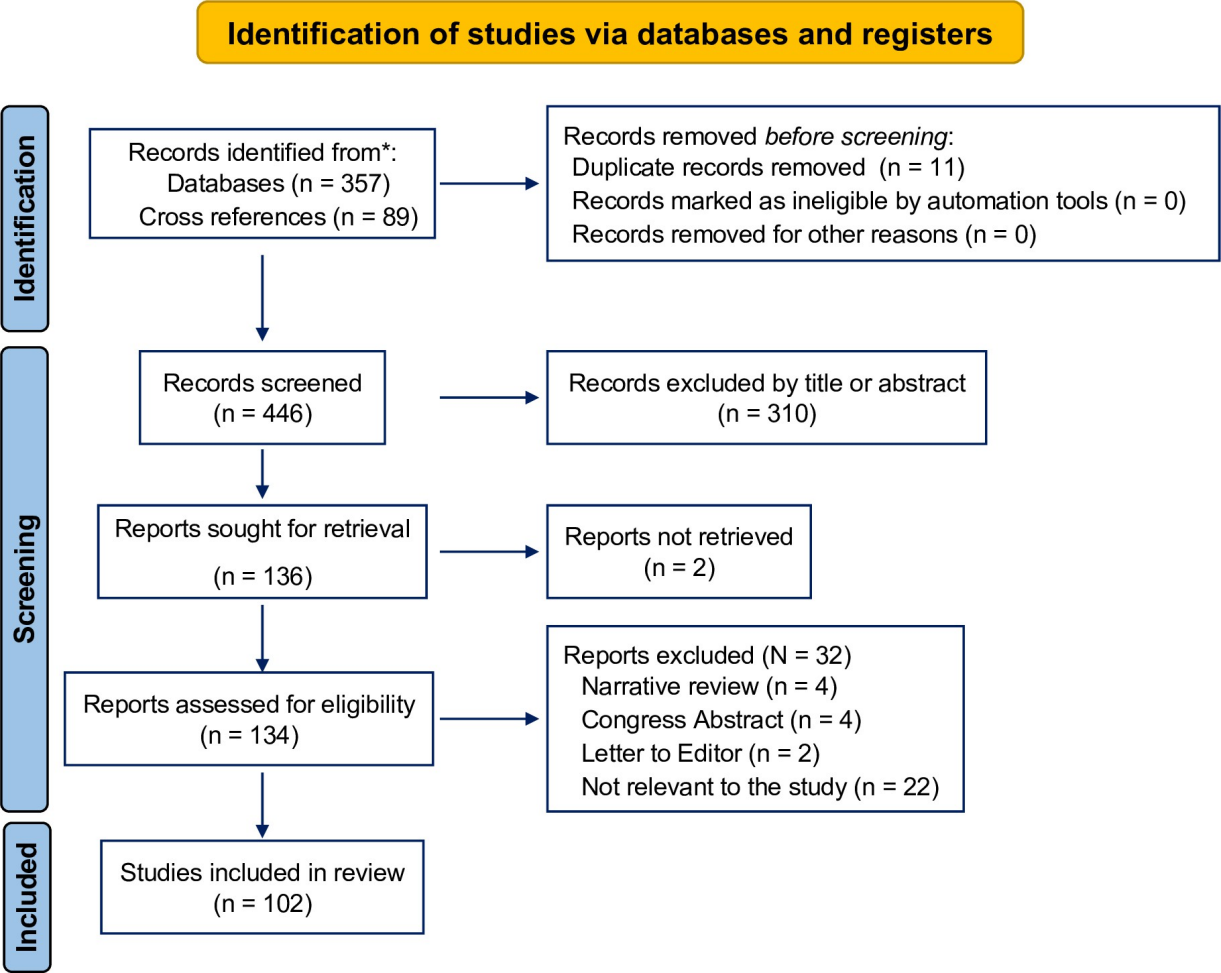
Diagram of pathogenic stages of cholangiohydatidosis. a) Hepatic cystic echinococcosis open into a bile duct. b) Incomplete cystic evacuation into the bile duct. c) Complete cystic evacuation into the bile duct.

In contrast, major CBCs, with an incidence of 3% to 17% of patients with CEL, are characterized by broad communication between the cyst wall and biliary tree. They are frequently detected preoperatively and typically during surgery. In these cases, the contents of the cyst can spontaneously drain into the bile duct, causing total or intermittent biliary obstruction [7], expressing itself clinically as biliary colic, obstructive jaundice and cholangitis [8]; (Fig 1). Such events can progress to sepsis, liver abscess formation, acute cholecystitis and even acute pancreatitis [9–14]. All of the above are situations that significantly increase the probability of developing postoperative complications [15].

Based on the pathogenic phenomena previously described, cholangiohydatidosis (CH) is defined as the presence of parasitic structures, characteristic of CEL (germ or pieces thereof, daughter vesicles or both), inside the bile duct, causing total or intermittent biliary obstruction, with or without secondary infection [8,16].

As such, it is an evolutionary complication of CEL, with a wide range of clinical manifestations that make preoperative diagnosis difficult at best. The reported prevalence is between 15.1% and 55.6%; and a cumulative incidence estimated at 0.07 cases in 5 years. It is furthermore associated with postoperative complications and in-hospital mortality of 23% and 7% respectively [15–18].

The aim of this study was to describe the available evidence regarding the clinical features of cholangiohydatidosis, postoperative complications, and hospital mortality.

## Methods

This manuscript was written following the PRISMA guideline statement (Preferred Reporting Items for Systematic Reviews and Meta-Analyses).

### Study protocol

PROSPERO (International prospective register of systematic reviews, NIHR) ID: CRD42023406867.

### Study design

A systematic review was conducted following the Updated guideline PRISMA statement [19].

### Eligibility criteria

Primary studies related to cholangiohydatidosis were included; without language restriction or year of publication. Editorials, letters to the editor, narrative reviews, consensus documents and discussions, and articles contaminated with patients infected with *E*. *multilocularis*, *E*. *vogelii*, or other parasites were excluded.

### Information sources

The following metasearch engines, libraries, and databases were reviewed: Trip Database, SciELO, BIREME-BVS, Web of Science (WoS), PubMed, EMBASE, and SCOPUS. The search and recruitment of articles closed on April 25, 2023.

### Search criteria

This was carried out using the PECO components (population study [P], exposure [E], comparator [C], and result [O]). Studies related to hepatic echinococcosis (P), with cholangiohydatidosis (E), without comparator (C), postoperative complications and hospital mortality (O). Sensitive searches were carried out using MeSH, DeCS and free terms (Echinococcosis; Cystic Echinococcosis; hydatidosis; echinococcus granulosus infection; Hepatic Hydatid Cyst; Liver Hydatid Cyst; Liver Hydatidosis; Hepatic hydatidosis; Cholangitis; cholangiohydatidosis; Hospital Mortality; mortality and Postoperative Complications); and boolean connectors (AND and OR), with strategies adapted to each database (Table 1). A manual and cross-reference search was also carried out.

**Table 1:** Search strategies and results obtained for each source of information used (N = 357).

*Meta search engines*, *libraries*, *and databases*	*Search strategies*
Trip Database (n = 4)	(echinococcosis OR "cystic echinococcosis" OR hydatidos* OR "echinococcus granulosus infection*" OR "infection*, echinococcus granulosus" OR "hepatic hydatid cyst" OR "liver hydatid cyst" OR "liver hydatidosis" OR "hepatic hydatidosis"), (cholangit* OR cholangiohydatidosis), ("hospital mortality" OR mortality OR "postoperative complications")
SciELO (n = 3)	(echinococcosis OR "Cystic Echinococcosis" OR hydatidos* OR "Echinococcus Granulosus Infection*" OR "Infection*, Echinococcus Granulosus" OR "Hepatic Hydatid Cyst" OR "Liver Hydatid Cyst" OR "Liver Hydatidosis" OR "Hepatic hydatidosis") AND (cholangit* OR cholangiohydatidosis)
WoS (n = 24)	(TS = (Echinococcosis OR "Cystic Echinococcosis" OR Hydatidos* OR "Echinococcus Granulosus Infection*" OR "Infection*, Echinococcus Granulosus" OR "Hepatic Hydatid Cyst" OR "Liver Hydatid Cyst" OR "Liver Hydatidosis" OR "Hepatic hydatidosis")) AND (TS = (Cholangit* OR Cholangiohydatidosis)) AND (TS = ("Hospital Mortality" OR Mortality OR "Postoperative Complications"))
PubMed (n = 44)	("Echinococcosis"[MeSH Terms] OR "Echinococcosis"[All Fields] OR "Cystic Echinococcosis"[All Fields] OR "hydatidos*"[All Fields] OR "echinococcus granulosus infection*"[All Fields] OR "infection echinococcus granulosus"[All Fields] OR "Hepatic Hydatid Cyst"[All Fields] OR "Liver Hydatid Cyst"[All Fields] OR "Liver Hydatidosis"[All Fields] OR "Hepatic hydatidosis"[All Fields]) AND ("Cholangitis"[MeSH Terms] OR "cholangit*"[All Fields] OR "cholangiohydatidosis"[All Fields]) AND ("Hospital Mortality"[MeSH Terms] OR "mortality"[MeSH Terms] OR "Postoperative Complications"[MeSH Terms])
EMBASE (n = 95)	(’liver hydatid cyst’/exp OR ’echinococcosis’/exp) AND ’cholangitis’/exp AND (’mortality’/exp OR ’hospital mortality’/exp OR ’postoperative complication’/exp)
SCOPUS (n = 117)	(TITLE-ABS-KEY(Echinococcosis OR "Cystic Echinococcosis" OR Hydatidos* OR "Echinococcus Granulosus Infection*" OR "Infection*, Echinococcus Granulosus" OR "Hepatic Hydatid Cyst" OR "Liver Hydatid Cyst" OR "Liver Hydatidosis" OR "Hepatic hydatidosis")) AND (TITLE-ABS-KEY(Cholangit* OR cholangiohydatidosis)) AND (TITLE-ABS-KEY("Hospital Mortality" OR Mortality OR "Postoperative Complications"))
Bireme BVS (n = 70)	((echinococcosis OR "Cystic Echinococcosis" OR hydatidos* OR "Echinococcus Granulosus Infection*" OR "Infection*, Echinococcus Granulosus" OR "Hepatic Hydatid Cyst" OR "Liver Hydatid Cyst" OR "Liver Hydatidosis" OR "Hepatic hydatidosis") AND ("Hospital Mortality" OR mortality OR "Postoperative Complications"))

### Study selection

Identified documents in each information source were filtered by duplication between databases. They were subsequently examined by title and abstract, applying eligibility criteria. The articles were then extensively analyzed by 3 reviewers (CM, JR and CR), all experienced in searching and analyzing biomedical studies. Discrepancy situations were resolved by consensus.

### Data collection

Critical review of each selected article, as well as the data extraction and its subsequent verification, was carried out by 3 researchers (CM, JR, and CR). Then, data was collected in an Excel spreadsheet (Mac Excel, version 15.24; 2016 Microsoft Corporation).

### Outcomes

Primary outcome variables were "postoperative complications", dichotomized in yes or no, classifying its according to Clavien & Dindo’s proposal [20], and “hospital mortality”, dichotomized in yes or no. Secondary outcome variables were year of publication, geographical origin of the studies, designs of primary studies, number of patients considered in each study, main clinical manifestation, anatomical location and type of cyst, hospital stay, a surgical procedure performed, reinterventions, and a different kind of associated therapeutics.

### Statistics

Descriptive statistics was applied with calculation of frequencies, averages, standard deviations and weighted averages.

### Additional analyses

**Fig 2:**
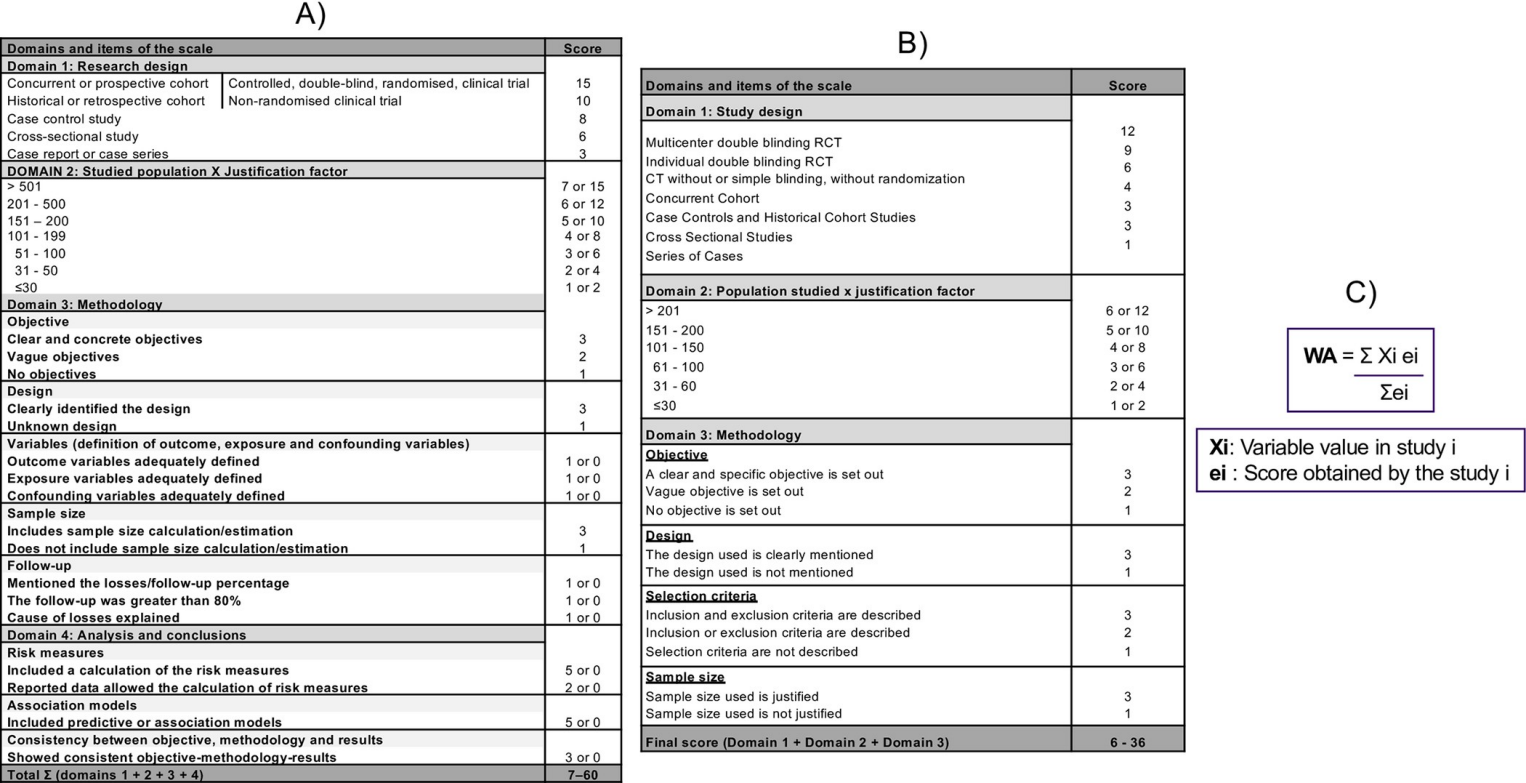
MInCir-T (A), MInCir-P (B), and weighted average formula (C), to determine methodological quality.

Meta-analysis was performed comparing the behavior of the variables under study in two periods of time (1982–2006, period A vs. 2007–2023, period B), applying a weighted least squares regression model (the weights were the number of patients of each scientific article). Methodological quality of primary studies was determined applying MInCir scales for therapeutic procedures and prognosis [21,22]. Dichotomization was decided because during the exploratory analysis of the data, we verified that this point divided the sample under study into two equal parts. Both used scales for determine methodologic quality (MQ), are valid (face and content validity, and construct validity for extreme groups) and reliable (interobserver reliability). MInCir-T scale, composed of 3 domains and 6 items: the first related to the study design; the second to the population sample size, and the third related to the methodology used; according to which, a score which represents the sum of the 3 domains is generated, with a final score that can vary between 6 and 36 points (6 points being the worst MQ study and 36 being the best), with a cut-off point to define the construct of 18 points [21]. And MInCir-P scale, composed of 4 domains and 11 items (study design, population sample size, methodology used, analysis and conclusions); according to which, a score which represents the sum of the 4 domains is generated, with a final score that can vary between 7 and 60 points (7 points being the worst MQ study and 60 being the best), with a cut-off point to define the construct of 33 points [22]. Then data were entered to a spreadsheet and calculation of weighted means was applied, and its comparison applying a weighted least squares regression model (Fig 2).

### Risk of bias in individual studies

The likelihood of inaccuracy in the estimate of causal effect in primary studies was assess applying MInCir scales [21,22], in both time periods.

### Ethics

In order to reduce selection and analysis biases masking of authors and study centers was implemented.

## Results

### Study selection

**Fig 3:**
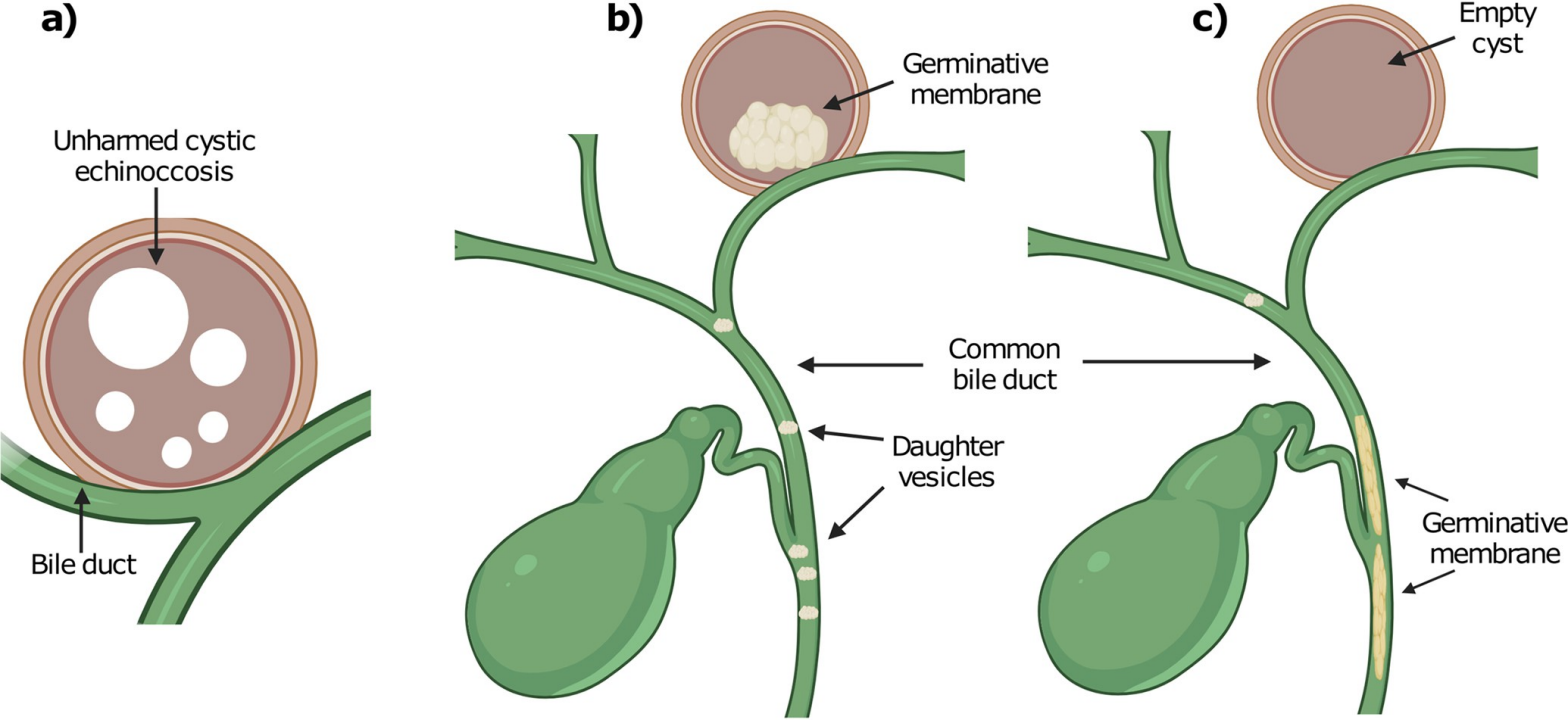
Flowchart of participating studies. Figure created with BIORENDER.COM, accessed on 30 November 2022.

357 studies were retrieved from the search, and 89 were obtained from cross-searches. Of these, 446 articles, 201 were eliminated due to duplication of information sources. 245 were examined by title and abstract, 109 of which were discarded because they were considered "not related" to the research; leaving 136 articles to evaluate eligibility by reading the full text (2 were not retrieved). An in-depth analysis of the selected studies was then performed, excluding 32 studies based on the review criteria. Finally, 102 studies correspond to the study sample of this review (Fig 3) [4,7,8,10,12,16,17,23,25–118].

### Study characteristics

39.2% of the included studies were published in the last decade (Table 2). Turkey had the highest number of publications (n = 29, 28.4%; Table 3). Most included studies were case reports and case series (64.7% and 33.3% respectively [Table 4]), representing 1241 patients, with a weighted mean age of 43.7 years, 50.6% male (Table 5).

**Table 2:** Year of publication of the included studies (N = 102).

*Year of publication*	*N° studies*	*%*
2018–2023	17	16.7
2013–2017	4	3.9
2012–2016	19	18.6
2007–2011	12	11.8
2002–2006	17	16.7
1997–2001	15	14.7
1992–1996	6	5.9
1987–1991	7	6.9
1982–1986	5	4.9
**T o t a l**	**102**	**100**

**Table 3:** Origen of included studies (N = 102).

*Study origin*	*N° studies*	*%*
Turkey	29	28.4
Greece	10	9.8
Spain	9	8.8
Morocco	9	8.8
Tunisia	7	6.9
India	6	5.9
Italy	5	4.9
Germany	3	2.9
Chile	3	2.9
Iran	3	2.9
Saudi Arabia	2	2.0
Israel	2	2.0
Pakistan	2	2.0
U.S.A	2	2.0
Others *	10	9.8
**T o t a l**	**102**	**100**

*: Belgium, China, Croatia, Netherlands, England, Libya, Moldova, Peru, Portugal, and Romania; one of each.

**Table 4:** Characteristics of the included studies and patients.

Variables	N° cases	%
Study design (n = 102)		
Case report	66	64.7
Case series	34	33.3
Historic cohort	2	2.0
Clinical presentation (n = 1244)		
Obstructive jaundice	639	51.4
Acute suppurative cholangitis	530	42.6
Acute pancreatitis	73	5.9
Cholangitis and acute pancreatitis	2	0.2
Findings in the bile duct (n = 102)		
Daughter vesicle	40	39.2
Germinative membrane	30	29.4
Both	32	31.4
Type of cysts, WHO classification (n = 102)		
CE2	31	30.4
CE3A	13	12.7
CE3B	41	40.3
CE4A	9	8.8
CE4B	8	7.8
Cholangiohydatidosis treatment (n = 1088)		
Choledocostomy with Kehr tube	669	61.5
Endoscopic surgery	292	26.8
Choledochoduodenum anastomosis	83	7.6
Suture of CQB	36	3.3
Percutaneous drainage	8	0.7
Cyst treatment (n = 1156)		
Partial cystectomy	713	61.7
Pericystectomy	216	18.7
Nothing	123	10.6
Liver resection	86	7.4
Cystostomy	18	1.6
Associated pharmacotherapy (n = 639)		
Albendazole	290	45.4
Antibiotics	272	42.6
Antibiotics + Albendazole	58	9.1
Mebendazole	16	2.5
Antibiotics + Mebendazole	3	0.5

**Table 5:** Clinical characteristics of patients and studies, and behavior of some variables in the two evaluated periods of time.

Variables	Weighted mean (N° studies = 102) (N° cases = 1241)	1982–2006 (N° studies = 52) (N° cases = 592)	2007–2023 (N° studies = 50) (N° cases = 649)	*p*
Age (years)	43.7±7.4	44.3±27.6	43.2±23.5	0.380
Postoperative complications (%)	26.2±19.1	25.6±63.1	27.0±81.2	0.732
Hospital mortality (%)	3.7±5.1	3.7±18.8	3.7±17.3	0.942
Re-interventions (%)	6.7±9.5	8.4±33.4	6.4±66.8	0.704
Total leukocytes (10e^3^/uL)	13208±4405	11407±14470	14540±7837	**0.016**
Total bilirubin (mg/dL)	5.5±3.5	5.1±9.6	5.9±7.1	0.403
Gamma-glutamyl transferase (U/L)	455±179	368±190	464±390	0.449
Alkaline phosphatase (U/L)	698±343	651±1060	743±669	0.406
ASAT (U/L)	263±232	295±811	241±197	0.420
ALAT (U/L)	301±170	270±613	328±253	0.271
Hospital stance (days)	14.4±6.3	13.5±20.1	16.1±24.6	0.107
Common bile duct diameter (mm)	16.3±6.7	20.3±15.8	13.8±15.3	**0.001**
Cysto-biliary communications (N°)	26.0±46.5	25.1±44.8	30.8±48.4	0.181
Cyst diameter (cm)	12.9±3.3	13.2±7.9	12.7±12.8	0.544
Follow-up (months)	67.9±51.0	70.6±154.8	66.2±141.9	0.791
MQ applying MInCir-T scale (points)	9.6±1.1	9.6±1.0	9.6±1.2	0.961
MQ applying MInCir-P scale (points)	12.9±3.3	14.2±2.3	14.8±5.7	0.918

**ASAT:** aspartate aminotransferase

**ALAT:** alanine aminotransferase

**MQ:** Methodological quality

### Synthesis of results

Weighted means of the common bile duct and cyst diameter were 16.3 mm and 12.9 cm, respectively (Table 5). Clinical presentation was obstructive jaundice, acute suppurative cholangitis, acute pancreatitis and a mixture of cholangitis with acute pancreatitis. In the common bile duct, daughter vesicles and germinative membrane were found. CE2 and CE3B were the most frequent types founded (Table 4). CH most frequent treatment was choledocostomy with a Kehr tube, and hydatid cyst’s most common treatment was partial cystectomy. Associated pharmacotherapy with benzimidazoles and antibiotics was utilized (Tables 5 and 6).

**Table 6:** Postoperative complications (n = 191).

*Postoperative complication*	*Clavien & Dindo*	*N° patients*	*%*
Surgical wound infection	I	32	16.8
Biliary fistula	IIIa	30	15.7
Biliary fistula	IIIb	21	11.0
Subphrenic abscess	IIIb	15	7.9
Atelectasis	II	14	7.3
Intra-abdominal abscess	IIIb	9	4.7
Infected residual cavity	IIIa	8	4.2
Pneumonia	IVa	8	4.2
Metabolic decompensation	IVa	6	3.1
Pleural empyema	IIIa	6	3.1
Sepsis	IVb	6	3.1
Bile duct stricture	IIIb	6	3.1
Bilioma formation	IIIb	5	2.6
Intestinal occlusion	IIIb	4	2.1
Evisceration	IIIb	4	2.1
Acute pancreatitis	IIIb	3	1.6
Cholangitis	IIIb	3	1.6
Atrial fibrillation	IIIa	3	1.6
Upper gastrointestinal bleeding	II	3	1.6
Infected residual cavity	IIIb	2	1.0
Anaphylaxis	IVb	2	1.0
Cyst rupture	IVa	1	0.5
**T o t a l**		**191**	**100**

Clavien I: 16.8%

Clavien II: 8.9%

Clavien III: 62.3% (IIIa: 24.6% and IIIb: 37.7%)

Clavien IV: 12.0% (IVa: 7.8% and IVb: 4.1%)

With a weighted mean of 14.4 days of hospital stance, it was verified that 26.2% of patients developed postoperative complications (74.3% Clavien and Dindo III and IV), 6.7% needed re-interventions, and 3.7% died (Table 5). The etiology and Clavien & Dindo distribution of postoperative complications is summarized in Table 6.

MQ of primary studies was 9.6±1.1 applying MInCir-Therapy scale (minimum 8 and maximum 15 points), and 14.5±4.3 applying MInCir-Prognosis scale (minimum 13 and maximum 53 points). When measuring methodological quality in the time periods under study, no statistical differences were observed (Table 5). Thus, the likelihood of inaccuracy in the estimate of causal effect in primary studies is high.

Finally, when comparing different in-study variables in two different time-periods, statistical difference in total leukocytes (*p* = 0.0163) and common bile duct diameter (*p* = 0.001) was observed. However, no differences were verified in postoperative complications, re-interventions or hospital mortality (Table 5).

### Possible biases in the review process

Additional information was requested from the authors to expand or verify some study data, unfortunately there was no response. Therefore, missing data may introduce bias in this review. The risk of missing studies was reduced by searching for cross references.

### Risk of bias between studies

There may be publication bias, given the concentration of studies from a few countries, and the paucity of studies from other geographic areas, places, and countries (Table 3).

## Discussion

### Summary of the evidence

This is the first SR that summarizes the available evidence concerning CH. There are one SR refers to gallbladder echinococcosis, based on 20 studies that include 22 cases [119].

The information of this SR was recovered from systematic searches carried out in 7 information sources (metasearch engines, libraries, and databases), including information of the last 40 years, and represents a compilation of 102 primary articles that represent 1241 patients treated in different countries, but preferably in Turkey, Greece, Morocco, and Spain.

The first drawback of the selection of MeSH terms and free words in order to define the search strategies, was the need to highlight that the CH concept is either unclear or is generally not well-known given that the number of publications found where CH appears in the title or abstract, is extremely limited (only 5 primary studies in this SR).

Most primary articles with which we worked had other types of denominations, such as “*frank biliary communication*”, “*frank intrabiliary rupture*”, “*frank cysto-biliary communication*”, “*ruptured liver hydatid cyst*”, “*opening in the biliary tract*”, “*complicated hepatic hydatid disease*”, etc., articulated with the concept "role of ERCP". Considering such, it is important to note that all these concepts, only define the existence of communications between a hydatid cyst and the bile duct, but do not consider necessarily the presence of hydatid material (daughter vesicles, germinal membrane, or a part thereof), inside the biliary tree. On the other hand, the use of different definitions for the same concept may represent a source of selection and information biases.

It was very interesting to note that in two different time periods, the variables of postoperative complications, re-interventions and hospital mortality showed very similar behavior, though without statistically significant differences. Consequently, it could be purported that the need for re-interventions, the morbidity and mortality associated with CH have not changed despite continuous advancements in technology, suggesting that CH is an aggressive disease itself.

In patients with sepsis due to cholangitis secondary to CH, who require emergency surgery, taking the patient directly to the operating room would seem indicated to repair the cysto-biliary communication, without previous ERCP, given that ERCP is an invasive procedure that should be performed by an experienced team, which is not always on site or available in emergency centers around the world [23].

On the other hand, is possible that type of cyst and or size has impact on the development of CH. For example, CE2 or CE3B type, has a higher rate of CH based on findings in bile duct (daughter vesicle and germinate membrane + daughter vesicle, reach 70.6%, because daughter cysts were present only in CE2 and CE3B). So, I it is possible could be a relationship between cyst size and/or type of cyst with CH.

Moreover, the variables that were found to be associated with the development of postoperative complications were as follows: common bile duct diameter (weighted average of 16.6 mm), main cyst diameter (weighted average of 13.2 cm), leukocytosis (weighted average of 13,469 10e3/uL), total bilirubin (weighted average of 5.5 mg/dL), alkaline phosphatase (weighted average of 799 U/L), and transaminases (weighted averages of 296 and 335 U/L).

In addition, the variables that were found to be associated with mortality were: common bile duct diameter (weighted average of 18.7 mm), main cyst diameter (weighted average of 13.8 cm), leukocytosis (weighted average of 14,546 10e3/uL), total bilirubin (weighted average of 4.8 mg/dL), alkaline phosphatase (weighted average of 621 U/L), and transaminases (weighted averages of 200 and 255 U/L).

We devised an alternative methodology for meta-analyzing data derived from studies of different design studies (especially in systematic reviews in which series of cases are the most frequent design founded in the searches). This approach involves the utilization of weighted averages, with weights assigned based on the methodological quality of the studies contributing to each datum [24].

### Limitations

The available evidence on CH was obtained only from level 4 studies (case reports, retrospective case series and historical cohorts, with reduced casuistry). On the other hand, methodological quality of primary studies was low: 9.6±1.1 for MInCir-Therapy and 14.5±4.3 for MInCir-Prognosis scales, which cutoff-points are 18 and 33 respectively [21,22].

### Conclusion

The available evidence related with CH is indicative that this evolutionary complication of EHQ is prone to severe postoperative complications, the need of reinterventions, and high hospital mortality rates, despite the passage of time.

## References

[1] Dolay K, Akbulut S. Role of endoscopic retrograde cholangiopancreatography in the management of hepatic hydatid disease. *World J Gastroenterol*. 2014;20:15253–61. doi: 10.3748/wjg.v20.i41.15253 25386073 PMC4223258

[2] Manterola C, Rojas C, Totomoch-Serra A, García-Méndez N, Riffo-Campos ÁL. Echinococcus granulosus genotypes verified in human hydatid disease around the world. Systematic review. *Rev Chilena Infectol*. 2020;37:541–9. 10.4067/S0716-10182020000500541. 33399801

[3] El Malki HO, El Mejdoubi Y, Souadka A, Mohsine R, Ifrine L, Abouqal R, Belkouchi A. Predictive model of biliocystic communication in liver hydatid cysts using classification and regression tree analysis. *BMC Surg*. 2010;10:16. doi: 10.1186/1471-2482-10-16 20398342 PMC2867769

[4] Zaouche A, Haouet K, Jouini M, El Hachaichi A, Dziri C. Management of liver hydatid cysts with a large biliocystic fistula: multicenter retrospective study. Tunisian Surgical Association. *World J Surg*. 2001;25:28–39. 10.1007/s002680020005. 11213153

[5] Akcan A, Sozuer E, Akyildiz H, Ozturk A, Atalay A, Yilmaz Z. Predisposing factors and surgical outcome of complicated liver hydatid cysts. *World J Gastroenterol*. 2010;16:3040–8. doi: 10.3748/wjg.v16.i24.3040 20572308 PMC2890945

[6] Manterola C, Vial M, Sanhueza A, Contreras J. Intrabiliary rupture of hepatic echinococcosis, a risk factor for developing postoperative morbidity: a cohort study. *World J Surg*. 2010;34:581–6. doi: 10.1007/s00268-009-0322-x 20087590

[7] Galati G, Sterpetti AV, Caputo M, Adduci M, Lucandri G, Brozzetti S, Bolognese A, Cavallaro A. Endoscopic retrograde cholangiography for intrabiliary rupture of hydatid cyst. *Am J Surg*. 2006;191:206–10. doi: 10.1016/j.amjsurg.2005.09.014 16442947

[8] Manterola C, Otzen T. Cholangiohydatidosis: an Infrequent Cause of Obstructive Jaundice and Acute Cholangitis. *Ann Hepatol*. 2017;16:436–41. doi: 10.5604/16652681.1235487 28425414

[9] Castillo S, Manterola C, Grande L, Rojas C. Infected hepatic echinococcosis. Clinical, therapeutic, and prognostic aspects. A systematic review. *Ann Hepatol*. 2021;22:100237. doi: 10.1016/j.aohep.2020.07.009 32835861

[10] Ben Mahmoud A, Atri S, Rebai W, Maghrebi H, Makni A, Kacem MJ. Acute pancreatitis as an uncommon complication of hydatid cyst of the liver: A case report and systematic literature review. *Ann Med Surg* *(Lond)*. 2021;62:341–6. doi: 10.1016/j.amsu.2021.01.079 33552493 PMC7847814

[11] Manterola C, Urrutia S; MINCIR GROUP. Infected Hepatic Echinococcosis: Results of Surgical Treatment of a Consecutive Series of Patients. *Surg Infect (Larchmt)*. 2015;16:553–7. doi: 10.1089/sur.2014.054 26125624

[12] Bektaş M, Dökmeci A, Cinar K, Halici I, Oztas E, Karayalcin S, et al. Endoscopic management of biliary parasitic diseases. *Dig Dis Sci*. 2010;55:1472–8. doi: 10.1007/s10620-009-0850-0 19513838

[13] Prousalidis J, Kosmidis C, Kapoutzis K, Fachantidis E, Harlaftis N, Aletras H. Intrabiliary rupture of hydatid cysts of the liver. *Am J Surg*. 2009;197:193–8. doi: 10.1016/j.amjsurg.2007.10.020 18558386

[14] Tomuş C, Iancu C, Pop F, Al Hajjar N, Puia C, Munteanu D, et al. Intrabiliary rupture of hepatic hydatid cysts: results of 17 years’ experience. *Chirurgia (Bucur)*. 2009;104:409–13. 19886047

[15] Manterola C, Urrutia S; Grupo MINCIR. Post surgery morbidity in patients with complicated hepatic hydatidosis. *Rev Chilena Infectol*. 2015;32:43–9. 10.4067/S0716-10182015000200010. 25860044

[16] Manterola C, Losada H, Carrasco R, Muñoz S, Bustos L, Vial M, Innocenti G. Cholangiohydatidosis. An evolutive complication of hepatic hydatidosis. *Bol Chil Parasitol*. 2001;56:10–5. 12058666

[17] Goumas K, Poulou A, Dandakis D, Tyrmpas I, Georgouli A, Sgourakis G, et al. Role of endoscopic intervention in biliary complications of hepatic hydatid cyst disease. *Scand J Gastroenterol*. 2007;42:1113–9. doi: 10.1080/00365520701234318 17710679

[18] Manterola C, Moraga J, Urrutia S. Aspectos clínico-quirúrgicos de la hidatidosis hepática, una zoonosis de creciente preocupación. *Rev Chil Cir*. 2011;63:641–9. 10.4067/S0718-40262011000600017.

[19] Page MJ, McKenzie JE, Bossuyt PM, Boutron I, Hoffmann TC, Mulrow CD, et al. The PRISMA 2020 statement: An updated guideline for reporting systematic reviews. *J Clin Epidemiol*. 2021;134:178–89. doi: 10.1016/j.jclinepi.2021.03.001 33789819

[20] Clavien PA, Barkun J, de Oliveira ML, Vauthey JN, Dindo D, Schulick RD, et al. The Clavien-Dindo classification of surgical complications: five-year experience. *Ann Surg*. 2009;250:187–96. doi: 10.1097/SLA.0b013e3181b13ca2 19638912

[21] Manterola C, Cartes-Velasquez R, Otzen T. Instructions for the Use of MInCir Scale to Assess Methodological Quality in Therapy Studies. *Int J Morphol*. 2015;33:1463–7. 10.4067/S0717-95022015000400045.

[22] Manterola C, Cartes-Velasquez R, Otzen T. Instructions for the Use of MInCiR Scale to Assess Methodological Quality in Prognosis Studies. *Int J Morphol*. 2015;33:1553–8. 10.4067/S0717-95022015000400059.

[23] Borahma M, Afifi R, Benelbarhdadi I, Ajana FZ, Essamri W, Essaid A. Endoscopic retrograde cholangiopancreatography in ruptured liver hydatid cyst. *Indian J Gastroenterol*. 2015;34: 330–4. doi: 10.1007/s12664-015-0585-0 26345677

[24] Manterola C, Vial M, Pineda V, Sanhueza A. Systematic review of literature with different types of designs. *Int J Morphol*. 2009;27:1179–86.

[25] Abou-Khalil S, Smith BM, MacLean JD, Poenaru D, Fried GM, Bret P. Acute cholecystitis and cholangitis caused by Echinoccocus granulosus. *Am J Gastroenterol*. 1996;91:805–7. 8677959

[26] Aghajanzadeh M, Ashoobi MT, Hemmati H, Samidoust P, Delshad MSE, Pourahmadi Y. Intrabiliary and abdominal rupture of hepatic hydatid cyst leading to biliary obstruction, cholangitis, pancreatitis, peritonitis and septicemia: a case report. *J Med Case Rep*. 2021;15:311. doi: 10.1186/s13256-021-02822-5 34049575 PMC8164221

[27] Ahmad BS, Afzal A, Ashraf P, Abubakar SA, Munir A. Manifestation of hydatid cyst of liver with pancreatitis, cholangitis and jaundice: A case report. *J Pak Med Assoc*. 2018;68:1097–9. 30317310

[28] Akaydin M, Erozgen F, Ersoy YE, Birol S, Kaplan R. Treatment of hepatic hydatid disease complications using endoscopic retrograde cholangiopancreatography procedures. *Can J Surg*. 2012;55:244–8. doi: 10.1503/cjs.036010 22617539 PMC3404144

[29] Akkis H, Akinoglu A, Colakoglu S, Demiryurek H, Yagmur O. Endoscopic management of biliary hydatid disease. *Can J Surg*. 1996;39:287–92. 8697318 PMC3950134

[30] Alhan E, Calik A, Küçüktülü U, Cìinel A. The intrabiliary rupture of hydatid cyst of the liver. *Nihon Geka Hokan*. 1994;63:3–9. 7826182

[31] Al Karawi MA, Yasawi MI, El-Shieck-Mohamed AR. Endoscopic management of biliary hydatid disease: Report of six cases. *Endoscopy*. 1991;23:278–81. 10.1055/s-2007-1010686. 1743129

[32] Alper A, Ariogul O, Emre A, Uras A, Okten A. Choledochoduodenostomy for intrabiliary rupture of hydatid cysts of liver. *Br J Surg*. 1987;74:243–5. doi: 10.1002/bjs.1800740405 3580792

[33] Al-Toma AA, Vermeijden RJ, Van De Wiel A. Acute pancreatitis complicating intrabiliary rupture of liver hydatid cyst. *Eur J Intern Med*. 2004;15:65–7. doi: 10.1016/j.ejim.2003.11.008 15066654

[34] Alvizuri-Escobedo JM, Sánchez-Mercado M. Cholangiohydatidosis. *Rev Soc Peru Med Interna*. 2011;24:149. http://medicinainterna.net.pe/revista/revista_24_3_2011/colangiohidatidosis.pdf.

[35] Arifuddin R, Baichi M, Ullah A, Maliakkal B. Cystic echinococcus—a rare presentation of acute biliary obstruction and pancreatitis. *J Clin Gastroenterol*. 2006;40:763–4. doi: 10.1097/00004836-200609000-00020 16940894

[36] Atli M, Kama NA, Yuksek YN, Doganay M, Gozalan U, Kologlu M, Daglar G. Intrabiliary rupture of a hepatic hydatid cyst: associated clinical factors and proper management. *Arch Surg*. 2001;136:1249–55. doi: 10.1001/archsurg.136.11.1249 11695968

[37] Avcu S, Ünal Ö, Arslan H. Intrabiliary rupture of liver hydatid cyst: a case report and review of the literature. *Cases J*. 2009;2:6455. doi: 10.1186/1757-1626-2-6455 19829807 PMC2709968

[38] Aydin A, Ersoz G, Tekesin O, Mentes A. Hydatid acute pancreatitis: a rare complication of hydatid liver disease. Report of two cases. *Eur J Gastroenterol Hepatol*. 1997;9:211–4. doi: 10.1097/00042737-199702000-00020 9058637

[39] Badea R, Badea G, Draƒühici A, Cazacu M, Porr PJ. Ultrasonic diagnosis of echinococcal cholangitis. *Ultraschall Med*. 1987;8:147–8. doi: 10.1055/s-2007-1011679 3310228

[40] Baraket O, Feki MN, Chaari M, Saidani A, Ben Moussa M, Moussa M, Bouchoucha S. Hydatid cyst open in biliary tract: therapeutic approaches. Report of 22 cases. J Visc Surg. 2011;148:e211–6. doi: 10.1016/j.jviscsurg.2011.05.009 21723216

[41] Basbous S, Hayette MP, Léonard P, Louis E, Loly JP, Detry O. Liver hydatidosis causing obstructive cholangitis: a case report. *Rev Med Liege*. 2021;76:575–8. . 34357705

[42] Bayhan Z, Yılmaz S, Tiryaki Ç, Kargı C, Ucar BI. Acute pancreatitis due to rupture of the hydatid cyst into the biliary tract: A case report. *NJMR*. 2014:2249–4995.

[43] Becker K, Frieling T, Saleh A, Haussinger D. Resolution of hydatid liver cyst by spontaneous rupture into the biliary tract. *J Hepatol*. 1997;2:1408–12. doi: 10.1016/s0168-8278(97)80479-5 9210631

[44] Belkouch A, Mouhsine A. Hydatid cyst ruptured in the biliary duct: an exceptional cause of acute pancreatitis. *Pan Afr Med J*. 2014;18:298. doi: 10.11604/pamj.2014.18.298.5147 25469191 PMC4247896

[45] Bellara IL, Amara H, Hablani N, Harzallah L, Abbassi DB, Kraiem C. Hydatic acute pancreatitis: a case report. *Ann Chir*. 2004;129:372–5. 10.1016/j.anchir.2004.04.017. 15297229

[46] Beltsis A, Chatzimavroudis G, Iliadis A, Tsavdaridis I, Vasiliadis I, Papaziogas B, Katsinelos T. Intrabiliary rupture of hepatic hydatid cyst presenting as acute pancreatitis and treatment with endoscopic sphincterotomy: report of two cases. *Ann*. *Gastroenterol*. 2005;18:353–6.

[47] Bilgi Kırmacı M, Akay T, Özgül E, Yılmaz S. Cholecysto-Hydatid Cyst Fistula: A Rare Cause of Cholangitis. *Am J Case Rep*. 2020;21:e921914. doi: 10.12659/AJCR.921914 32546677 PMC7319074

[48] Busić Z, Amić E, Servis D, Predrijevac M, Stipancić I, Busić D. Common bile duct obstruction caused by the hydatid daughter cysts. *Coll Antropol*. 2004;28:325–9. 15636090

[49] Cakır OO, Ataseven H, Demir A. Hydatid acute pancreatitis. *Turkiye Parazitol Derg*. 2012;36:251–3. 10.5152/tpd.2012.59. 23339949

[50] Chaudhary A, Upadhyaya AC, Kankanala VV, Kumar A, Joshi N, Kumar M, et al. Intrabiliary rupture of hepatic hydatid cyst with impacted hydatid membranes at ampulla of Vater presenting as acute pancreatitis. *Trop Gastroenterol*. 2013;34:43–4. doi: 10.7869/tg.2012.93 23923377

[51] Cicek B, Parlak E, Disibeyaz S, Oguz D, Cengiz C, Sahin B. Endoscopic therapy of hepatic hydatid cyst disease in preoperative and postoperative settings. *Dig Dis Sci*. 2007;52:931–5. doi: 10.1007/s10620-006-9426-4 17333353

[52] Coşkun O, Ödemiş B, Alpua M, et al. Endoscopic cyst evacuation as a treatment for liver hydatic cysts in patients who present with acute pancreatitis. *Endosc Gastrointest*. 2017;25:49–51. 10.17940/endoskopi.339876.

[53] Daali M, Fakir Y, Hssaida R, Hajji A, Had A. Hydatid cysts of the liver opening in the biliary tract. Report of 64 cases. *Ann Chir*. 2001;126:242–5. 10.1016/s0003-3944(01)00507-7. 11340710

[54] Dadoukis J, Gamvros O, Aletras H. Intrabiliary rupture of the hydatid cyst of the liver. *World J Surg*. 1984;8:786–90. doi: 10.1007/BF01655782 6506741

[55] Daldoul S, Ben Dhaou A, Ben Tahar A, Baccari A, Khemir A, Ben Safta Y, Ben Moussa M. Internal transfistulary drainage for intrabiliary rupture of hydatid cyst of the liver: Analysis of the indications and the results. Report of 50 cases. *Tunis Med*. 2017;95:10–8. 29327763

[56] Davidson B, Ezaki T, Habib N. Controversy in the management of cholangitis secondary to hydatid daughter cysts. *HPB Surg*. 1991;4:321–9. doi: 10.1155/1991/58262 1810374 PMC2423643

[57] Dumas R, Le Gall P, Hastier P, Buckley MJ, Conio M, Delmont JP. The role of endoscopic retrograde cholangiopancreatography in the management of hepatic hydatid disease. *Endoscopy*. 1999;31:242–7. doi: 10.1055/s-1999-14209 10344429

[58] Elbir O, Gundogdu H, Caglikulekci M, Kayaalp C, Atalay F, Savkilioglu M, Seven C. Surgical treatment of intrabiliary rupture of hydatid cysts of liver: comparison of choledochoduodenostomy with T-tube drainage. *Dig Surg*. 2001;18:289–93. doi: 10.1159/000050154 11528138

[59] el Idrissi HD, Ridai M, Zerouali NO. Pancreatitis of hydatid origin. *Presse Med*. 1996;25:2022–4. 9082376

[60] Ghidirim, Mişin I, Guţu E, Gagauz I, Danci A, Vozian M, Zastavniţchi GH. Intrabiliary rupture of the hydatic cyst complicated with acute pancreatitis. *Chirurgia (Bucur)*. 2006;101:429–32. 17059157

[61] Giordano G, Ialongo P, Amoruso M, Rizzo MI, Di Venere B, Bonomo GM. Obstructive jaundice caused by hydatid cyst rupture in main bile duct. *Ann Ital Chir*. 1999;70:763–6. 10692798

[62] Giouleme O, Nikolaidis N, Zezos P, Budas K, Katsinelos P, Vasiliadis T, Eugenidis N. Treatment of complications of hepatic hydatid disease by ERCP. *Gastrointest Endosc*. 2001;54:508–10. doi: 10.1067/mge.2001.118256 11577320

[63] González-Arboleda F, Pizarro F, Lindner C, Caqueo F. Therapeutic approach of complicated hydatid disease: role of endoscopic retrograde cholangiopancreatography in cholangiohydatidosis. *Arq Bras Cir Dig*. 2023;35:e1699. doi: 10.1590/0102-672020220002e1699 36629682 PMC9831634

[64] Hafeez M, Hussain T, Salamat A, Hassan F, Farooq A, Saeed F. Ruptured hydatid cyst in biliary tract. *J Coll Physicians Surg Pak*. 2012;22:663–5. doi: 10.2012/JCPSP.663665 23058154

[65] Jaén-Torrejimeno I, Latorre-Fragua R, López-Guerra D, Rojas-Holguín A, Manuel-Vázquez A, Blanco-Fernández G, Ramia JM. Jaundice as a clinical presentation in liver hydatidosis increases the risk of postoperative biliary fistula. *Langenbecks Arch Surg*. 2021;406:1139–47. doi: 10.1007/s00423-020-02070-z 33389115

[66] João M, Silva M, Perdigoto D, Mendes S. Cholangiohydatidosis: an uncommon cause of acute cholangitis. *Rev Esp Enferm Dig*. 2020;112:881–2. doi: 10.17235/reed.2020.7063/2020 33054284

[67] Katsinelos P, Chatzimavroudis G, Fasoulas K, Kamperis E, Katsinelos T, Terzoudis S, et al. Acute pancreatitis caused by impaction of hydatid membranes in the papilla of Vater: a case report. *Cases J*. 2009;2:1–4. 10.4076/1757-1626-2-7374. 19829949 PMC2740072

[68] Khoshbaten M, Farhang S, Hajavi N. Endoscopic retrograde cholangiography for intrabiliary rupture of hydatid cyst. *Dig Endosc*. 2009;21:277–9. doi: 10.1111/j.1443-1661.2009.00907.x 19961531

[69] Kitchens WH, Liu C, Ryan ET, Fernandez-del Castillo C. Hepatic hydatid cyst: a rare cause of recurrent pancreatitis. *J Gastrointest Surg*. 2014;18:2057–9. doi: 10.1007/s11605-014-2630-1 25149853

[70] Kırmacı MB, Akay T, Özgül E, Yılmaz S. Cholecysto-Hydatid Cyst Fistula: A Rare Cause of Cholangitis. *Am J Case Rep*. 2020;21:e921914. doi: 10.12659/AJCR.921914 32546677 PMC7319074

[71] Koksal N, Muftuoglu T, Gunerhan Y, Uzun MA, Kurt R. Management of intrabiliary ruptured hydatid disease of the liver. *Hepatogastroenterology*. 2001;48:1094–6. 11490808

[72] Kornaros SE, Aboul-Nour TA. Frank intrabiliary rupture of hydatid hepatic cyst: diagnosis and treatment. *J Am Coll Surg*. 1996;183:466–70. 8912615

[73] Lahmidani FZ, Hamdoun H, Abid M, El Yousfi DA, Benajah M, El Abkari SA, Ibrahimi. Acute pancreatitis as a rare complication of rupture of hydatid liver cyst: review of 16 cases. *PAMJ Clin Med*. 2019;1:1–5. 10.11604/pamj-cm.2019.1.17.20944.

[74] Mahmoudi A, Zouari K. A rare complication of hydatid cyst of the liver: acute pancreatitis. *Pan Afr Med J*. 2015;21:247. 10.11604/pamj.2015.21.247.7600. 26523186 PMC4607990

[75] Manouras A, Genetzakis M, Antonakis PT, Lagoudianakis E, Pattas M, Papadima A, et al. Endoscopic management of a relapsing hepatic hydatid cyst with intrabiliary rupture: a case report and review of the literature. *Can J Gastroenterol*. 2007;21:249–53. doi: 10.1155/2007/410308 17431515 PMC2657701

[76] Mathur SK, Shah RR, Samsi AB, Kelkar MD. Hydatid cyst as a cause of common bile duct obstruction (a case report). *J Postgrad Med*. 1983;29:262–6. https://www.jpgmonline.com/text.asp?1983/29/4/262/5502. 6672187

[77] McCorkell SJ. Echinococcal cysts in the common bile duct: an uncommon cause of obstruction. *Gastrointest Radiol*. 1985;10:390–3. doi: 10.1007/BF01893138 4054507

[78] Medina E, Orti E, Canelles P, Calvo MA, Molina E. Complicated hepatic hydatid cyst and acute pancreatitis. Value of ERCP and treatment with endoscopic sphincterotomy. *Rev Esp Enferm Dig*. 1990;78:315–8. 2090177

[79] Menteş A, Batur Y, Eldem A, Ozbal O. Pancreatitis as a complication of a hydatic liver cyst—a case report. *Jpn J Surg*. 1990;20:356–8. doi: 10.1007/BF02470674 2359211

[80] Michalopoulos N, Laskou S, Papavramidis TS, Pliakos I, Kotidis E, Kesisoglou I, Papavramidis ST. Rupture of right hepatic duct into hydatid cyst. *J Korean Med Sci*. 2012;27:953–6. doi: 10.3346/jkms.2012.27.8.953 22876065 PMC3410246

[81] Moujahid M, Tajdine MT. Hydatid cysts of the liver ruptured into the biliary tracts: report of 120 cases. *Pan Afr Med J*. 2011;10:43. 22384289 PMC3290873

[82] Naranjo A, Sánchez del Río A, Vignote L, Vega JF, Mino G. Effectiveness of endoscopic sphincterotomy in complicated hepactic hydatid disease. *Gastrointest Endosc*. 1998;48:593–7. 10.1016/s0016-5107(98)70041-0. 9852449

[83] Nemati Honar B, Hayatollah G, Nikshoar M, Forootan M, Feizi AM. Liver Hydatid Cyst and Acute Cholangitis: a Case Report. *Acta Med Iran*. 2016;54:286–8. 27309273

[84] Ochando Cerdán F. Ictericia obstructiva y colangitis secundaria a quiste hidatídico hepático. *Cir Esp*. 2006;80:53–6. 16796958 10.1016/s0009-739x(06)70920-4

[85] Önder RO, Bekci T, Yayla Dİ. Ruptured hepatic hydatid cyst causing cholangitis. *Rev Soc Bras Med Trop*. 2022;55:e0530–2022. doi: 10.1590/0037-8682-0530-2022 36542025 PMC9757702

[86] Ovnat A, Peiser J, Avinoah E, Barki Y, Charuzi I. Acute cholangitis caused ruptured hydatid cyst. *Surgery*. 1984;5:497–500. 6710345

[87] Ozcaglayan O, Halefoglu AM, Ozcaglayan T, Sumbul HA. Ultrasonographic diagnosis of acute pancreatitis caused by ruptured hydatid disease to the biliary system. *BR-BTR*. 2014;97:33–5. 10.5334/jbr-btr.8. 24765770

[88] Paksoy M, Karahasanoglu T, Carkman S, Giray S, Senturk H, Ozcelik F, Erguney S. Rupture of the hydatid disease of the liver into the biliary tracts. *Dig Surg*. 1998;15:25–9. doi: 10.1159/000018582 9845559

[89] Parthé S, Maier M, Kohler B, Kress S, Riemann JF. Acute pancreatitis due to the rupture of an echinococcal cyst into the bile duct system. *Dtsch Med Wochenschr*. 1994;119:624–7. 10.1055/s-2008-1058739. 7513277

[90] Pavlidis TE, Katsinelos PT, Tsiaousis PZ, Atmatzidis KS. Intrabiliary rupture of a large liver echinococcal cyst in an adolescent managed with endoscopic sphincterotomy and albendazole. *J Laparoendosc Adv Surg Tech A*. 2006;16:493–6. doi: 10.1089/lap.2006.16.493 17004876

[91] Ramia Angel JM, Sancho Calatrava E, Veguillas Redondo P, Santos Blanco JM, García-Parreño Jofré J. Acute hydatid pancreatitis. *Rev Esp Enferm Dig*. 2008;100:733–4. 10.4321/s1130-01082008001100015. 19159182

[92] Robleh H, Yassine F, Driss K, Khalid E, Fatima-Zahra B, Saad B, Rachid L, Abdalaziz F, Najib ZO. Total rupture of hydatid cyst of liver in to common bile duct: a case report. *Pan Afr Med J*. 2014;19:370. doi: 10.11604/pamj.2014.19.370.5727 25932083 PMC4407934

[93] Rodríguez-Sicilia MJ, Gonzalez-Artacho C, Cabello-Tapia MJ, de-la-TorreRubio P, de-Teresa-Galvan J. Recurrent acute pancreatitis as clinical presentation of hydatid disease of the liver. *Rev Esp Enferm Dig*. 2012;104:441–2. 10.4321/S1130-01082012000800011. 23039808

[94] Saez-Royuela F, Yuguero L, López-Morante A, Pérez-Alvarez JC, Martín-Lorente JL, Ojeda C. Acute pancreatitis caused by hydatid membranes in the biliary tract: Treatment with endoscopic sphincterotomy. *Gastrointest Endosc*. 1999;49:793–6. doi: 10.1016/s0016-5107(99)70305-6 10343232

[95] Settaf A, Bargach S, Aghzadi R, Lahlou MK, Oudghiri M. Treatment of cystobiliary fistula of hydatid cyst of the liver. Apropos of 33 cases. *J Chir (Paris)*. 1991;128:133–8. 2055975

[96] Schwartz MP, Samsom M. Image of the month. Ruptured Echinococcal cyst causing acute cholangitis. *Gastroenterology*. 2001;121:1039. doi: 10.1053/gast.2001.29352 11706829

[97] Sciumè C, Geraci G, Pisello F, Li Volsi F, Facella T, Modica G. Treatment of complications of hepatic hydatid disease by ERCP: our experience. *Ann Ital Chir*. 2004;75:531–5. 15960339

[98] Sciumè C, Geraci G, Pisello F, Li Volsi F, Facella T, Modica G. Acute pancreatitis during liver hydatidosis: treatment with ERCP and endoscopic sphincterotomy. *Ann Ital Chir*. 2005;76:491–4. 16696226

[99] Shalayiadang P, Jiang T, Yimiti Y, Ran B, Aini A, Zhang R, et al. Double versus single T-tube drainage for frank cysto-biliary communication in patients with hepatic cystic echinococcosis: a retrospective cohort study with median 11 years follow-up. *BMC Surg*. 2021;21:12. 10.1186/s12893-020-01028-8. 33407348 PMC7789643

[100] Sharma BC, Reddy RS, Garg V. Endoscopic management of hepatic hydatid cyst with biliary communication. *Dig Endosc*. 2012;24:267–70. doi: 10.1111/j.1443-1661.2011.01225.x 22725113

[101] Shemesh E, Klein E, Abramowich D, Pines A. Common bile duct obstruction caused by hydatid daughter cysts—management by endoscopic retrograde sphincterotomy. *Am J Gastroenterol*. 1986;81:280–2. 3962953

[102] Sıkar HE, Kaptanoglu L, Kement M. An unusual appearance of complicated hydatid cyst: necrotizing pancreatitis. *Ulus Travma Acil Cerrahi Derg*. 2017;23:81–3. doi: 10.5505/tjtes.2016.26820 28261778

[103] Simsek H, Ozaslan E, Sayek I, Sava≈ü C, Abbasoƒülu O, Soylu AR, et al. Diagnostic and therapeutic ERCP in hepatic hydatid disease. *Gastrointest Endosc*. 2003;58:384–9. doi: 10.1067/s0016-5107(03)00013-0 14528213

[104] Singh V, Reddy DC, Ram Verma G, Singh G. Endoscopic management of intrabiliary-ruptured hepatic hydatid cyst. *Liver Int*. 2006;26:621–4. doi: 10.1111/j.1478-3231.2006.01259.x 16762008

[105] Spârchez Z, Osian G, Onica A, Bărbântă C, Tanţău M, Pascu O. Ruptured hydatid cyst of the liver with biliary obstruction: presentation of a case and review of the literature. *Rom J Gastroenterol*. 2004;13:245–50. 15470540

[106] Tacyildiz I, Aldemir M, Aban N, Keles C. Diagnosis and surgical treatment of intrabiliary ruptured hydatid disease of the liver. *S Afr J Surg*. 2004;42:43–6. 15253319

[107] Tekin A, Küçükkartallar T, Aksoy F, Metin Belviranlı, Murat Çakır, Bülent Erenoğlu. Our patients with hepatic hydatid cyst rupturing into bile ducts. *Turk J Surg*. 2007;23:125–8.

[108] Toka B, Karaman K, Eminler AT, et al. A Case of Cyst Hydatid which is Diagnosed By Appperarence of Girl Vesicles in Choledoc with ERCP. *Sakarya Tıp Dergisi*. 2016;6:240–4. 10.5505/sakaryamedj.2016.33602.

[109] Toumi O, Ammar H, Gupta R, Ben Jabra S, Hamida B, Noomen F, et al. Management of liver hydatid cyst with cystobiliary communication and acute cholangitis: a 27-year experience. *Eur J Trauma Emerg Surg*. 2019;45:1115–9. doi: 10.1007/s00068-018-0995-7 30191292

[110] Trébol J, Sánchez R, Blanco CA, Rodríguez MB, Roldán M, Ozalla F, Francos CM. Quiste hidatídico hepático con migración de material hidatídico a la vía biliar principal, colangitis y pancreatitis aguda. *Revista ACIRCAL*. 2013. http://www.acircal.net/revista/articulo.php?id=17.

[111] Trigui A, Rejab H, Krichene J, Tlili A, Trabelsi J, Kachoua A, et al. Surgical treatment of hydatid cysts with large biliocystic fistula in conservative strategy. *Pan Afr Med J*. 2021;38:195. 10.11604/pamj.2021.38.195.27098. 33995801 PMC8106792

[112] Turan M, Duman M, Aydin C, Erdem M, Goktas S, Topcu O, et al. Management of frank intrabiliary rupture of hepatic hydatid cyst. *Chirurgische Gastroenterologie*. 2005;21:74–7. doi: 10.1159/000082675

[113] Ufuk F, Duran M. Intrabiliary Rupture of Hepatic Hydatid Cyst Leading to Biliary Obstruction, Cholangitis, and Septicemia. *J Emerg Med*. 2018;54:e15–e17. doi: 10.1016/j.jemermed.2017.09.009 29107478

[114] Ugurlu ET, Baykan M, Donder Y. Treatment of hepatic hydatid cyst rupture into the biliary tract: endoscopic evacuation. *Acta Medica Nicomedia*. 2022;5:218–22. 10.53446/actamednicomedia.1137988.

[115] Villán-González A. Obstructive jaundice secondary to a hepatic hydatid cyst. *Rev Esp Enferm Dig*. 2018:110:741–2. doi: 10.17235/reed.2018.5574/2018 30238755

[116] Wani I, Bhat Y, Khan N, Mir F, Nanda S, Shah OJ. Concomitant Rupture of Hydatid Cyst of Liver in Hepatic Duct and Gallbladder: Case Report. *Gastroenterology Res*. 2010;3:175–9. doi: 10.4021/gr215e 27942301 PMC5139739

[117] Yahya AI, Shwereif HE, Thoboot AS, Ekheil MA, Algader KA, Abosherda A, et al. Hepatic Hydatid Induced Obstructive Jaundice. *Ann Clin Pathol*. 2016;4(1):1059.

[118] Yücesoy AN, Poçan S. Secondary gallbladder hydatidosis and nonfragmanted germinative membrane sourced obstructive jaundice caused by intrabiliary ruptured hepatic hydatid cyst (a case report): two rare complication of the intrabiliary ruptured hepatic hydatid cyst. *Hepatobiliary Surg Nutr*. 2014;3:209–11. doi: 10.3978/j.issn.2304-3881.2014.07.05 25202699 PMC4141288

[119] Yagnik VD, Dawka S, Patel N. Gallbladder Hydatid Cyst: A Review on Clinical Features, Investigations and Current Management. *Clin Exp Gastroenterol*. 2020;13:87–97. doi: 10.2147/CEG.S243344 32308464 PMC7135162

